# A real-world disproportionality analysis of Tivozanib data mining of the public version of FDA adverse event reporting system

**DOI:** 10.3389/fphar.2024.1408135

**Published:** 2024-06-13

**Authors:** Kaixuan Wang, Mengmeng Wang, Wensheng Li, Xiaohui Wang

**Affiliations:** ^1^ Department of Urology Surgery, The First Affiliated Hospital, and College of Clinical Medicine of Henan University of Science and Technology, Luoyang, China; ^2^ Department of Oncology, the Second Affiliated Hospital of Henan University of Science and Technology, Luoyang, China

**Keywords:** FAERS, Tivozanib, adverse events, ccRCC, adverse drug reactions

## Abstract

**Background:**

Tivozanib, a vascular endothelial growth factor tyrosine kinase inhibitor, has demonstrated efficacy in a phase III clinical trials for the treatment of renal cell carcinoma. However, comprehensive evaluation of its long-term safety profile in a large sample population remains elusive. The current study assessed Tivozanib-related adverse events of real-world through data mining of the US Food and Drug Administration Adverse Event Reporting System FDA Adverse Event Reporting System.

**Methods:**

Disproportionality analyses, utilizing reporting odds ratio proportional reporting ratio Bayesian confidence propagation neural network and multi-item gamma Poisson shrinker (MGPS) algorithms, were conducted to quantify signals of Tivozanib-related AEs. Weibull distribution was used to predict the varying risk incidence of AEs over time.

**Results:**

Out of 5,361,420 reports collected from the FAERS database, 1,366 reports of Tivozanib-associated AEs were identified. A total of 94 significant disproportionality preferred terms (PTs) conforming to the four algorithms simultaneously were retained. The most common AEs included fatigue, diarrhea, nausea, blood pressure increased, decreased appetite, and dysphonia, consistent with prior specifications and clinical trials. Unexpected significant AEs such as dyspnea, constipation, pain in extremity, stomatitis, and palmar-plantar erythrodysaesthesia syndrome was observed. The median onset time of Tivozanib-related AEs was 37 days (interquartile range [IQR] 11.75–91 days), with a majority (n = 127, 46.35%) occurring within the initial month following Tivozanib initiation.

**Conclusion:**

Our observations align with clinical assertions regarding Tivozanib’s safety profile. Additionally, we unveil potential novel and unexpected AE signatures associated with Tivozanib administration, highlighting the imperative for prospective clinical studies to validate these findings and elucidate their causal relationships. These results furnish valuable evidence to steer future clinical inquiries aimed at elucidating the safety profile of Tivozanib.

## 1 Introduction

Renal cell carcinoma (RCC) manifests as malignancy originating from renal epithelium, characterized by its subdivision into more than ten distinct molecular subtypes delineated by histological and molecular attributes (Hsieh et al., 2017). Among these subtypes, clear cell RCC (ccRCC) stands as the most prevalent, contributing substantially to kidney cancer mortality rates (Jonasch et al., 2021). Despite advancements in early diagnosis and successful management via surgical or ablative interventions, the emergence of metastases remains a significant challenge for most ccRCC patients (Jonasch et al., 2021). The therapeutic landscape has witnessed notable evolution with the advent of various inhibitors targeting vascular endothelial growth factor (VEGF) and HIF2α, often employed alone or in combination with immune-checkpoint blockers (Choueiri and Kaelin, 2020). Recent therapeutic strides have introduced novel treatment modalities within the realm of tyrosine kinase inhibitors (TKIs), including agents such as nivolumab, cabozantinib, lenvatinib, and Tivozanib (Motzer et al., 2015a; Motzer et al., 2015b; Choueiri et al., 2016; Zarrabi et al., 2017).

Tivozanib, a potent tyrosine kinase inhibitor (TKI), received its inaugural approval from the Food and Drug Administration (FDA) in 2021 (Chang et al., 2022). Noteworthy outcomes from the phase III TIVO-3 trial among patients with advanced ccRCC receiving Tivozanib demonstrated significant enhancements in both survival and objective response rates (Rini et al., 2020; Beckermann et al., 2024). Comparative analyses against sorafenib underscored superior treatment efficacy with Tivozanib administration, resulting in prolonged survival durations (Johns et al., 2024). However, despite the apparent reduction in adverse events (AEs) associated with Tivozanib compared to sorafenib, the occurrence of TKI-related AEs remains notable among patient reports (Rini et al., 2020; Xie et al., 2023; Chen et al., 2024; Johns et al., 2024). Consequently, the imperative to comprehensively analyze and mitigate Tivozanib-associated AEs warrants urgent attention before its clinical application.

The FDA Adverse Event Reporting System (FAERS) stands as a publicly accessible spontaneous reporting repository aggregating millions of AEs submitted by diverse stakeholders including physicians, pharmacists, and manufacturers (Cohen et al., 2000; Rahimi et al., 2009). Esteemed as the world’s largest pharmacovigilance database, FAERS has demonstrated efficacy in detecting adverse drug reactions (ADRs) associated with various pharmaceutical interventions (Shu et al., 2022a; Shu et al., 2022b). The spontaneous repoeting system has been utilized in pharmacovigilance for safety assessment suspected AEs and plays a major part in signal identification due to inherent limitations of clinical trials such as stringent trial design, strict enrollment criteria, relatively small sample size and limited follow-up duration (Gupta and Fishman, 2011; Albiges et al., 2021; Guo et al., 2022). In this investigation, we retrospectively scrutinize AEs linked to Tivozanib utilization as reported through FAERS data mining spanning the period between January 2021 and December 2023.

## 2 Materials and methods

### 2.1 Study design and data sources

FAERS, recognized as a cornerstone for post-marketing safety surveillance, serves as a comprehensive repository for AE reports sourced from diverse stakeholders including healthcare professionals, pharmaceutical manufacturers, individual patients, and others. The FAERS dataset encompasses seven distinct components: DEMO (comprising patient demographic and administrative details), DRUG (housing drug-specific information), REAC (coded representations of reported adverse events), OUTC (reflecting patient outcomes), RPSR (indicating sources of reports), THER (documenting therapy initiation and cessation dates for reported drugs), and INDI (outlining indications for drug administration), along with a category for deleted cases. Each dataset sourced from the U.S. FDA website underwent integration into the R software platform (version 4.3.1) to facilitate subsequent analysis. This study leveraged the FAERS database, extracting data spanning the timeframe from January 2021 to December 2023.

**FIGURE 1:**
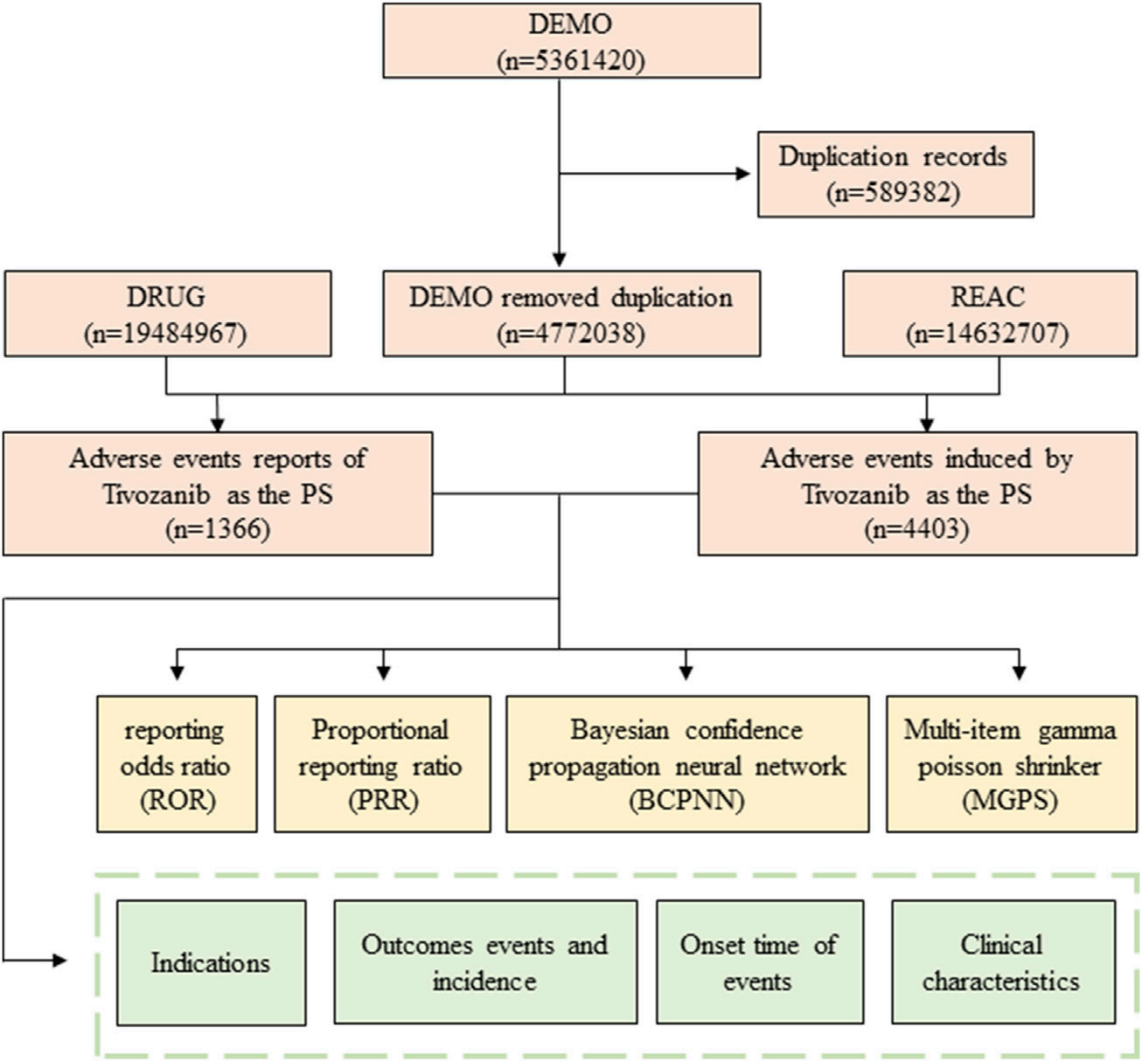
Flowchart of identifying adverse event cases of Tivozanib and statins from the FAERS database.

After the raw data collection, we identified and corrected errors, inconsistencies, and missing values to ensure the quality of the data for analysis. A comprehensive flowchart outlining the meticulous processes of data extraction, processing, and analysis has been meticulously depicted in Figure 1.

The terminology framework of the Medical Dictionary for Regulatory Activities (MedDRA) is structured into five hierarchical levels: system organ class (SOC), high-level group term (HLGT), high-level term (HLT), preferred term (PT), and lowest-level term (LLT) (Burr et al., 2019). AEs are coded using PTs according to the MedDRA (version 25.0) in FAERS. In the current study, each AE reported in association with Tivozanib was mapped to PT and SOC level based on the structural hierarchy of the MedDRA terminology (Cammà et al., 1997). Drugs identified within FAERS were categorized into four distinct classifications: PS (primary suspect), SS (secondary suspect), C (concomitant), and I (interacting). We identified cases using generic names (Tivozanib) and trade names (Fotivda) and selected role_cod as the PS to improve accuracy. Noteworthy patient outcomes encompassed critical indicators such as death (DE), life-threatening events (LT), initial or prolonged hospitalization (HO), disability (DS), congenital anomalies (CA), or other medically significant occurrences (OT) (Steinhart et al., 1994). In addition to AEs data, pertinent clinical features including gender, age, reporting region, reporter identity, and reporting duration were meticulously collected and described (Louis et al., 2022).

### 2.2 Statistical analysis

An analytical description was employed to depict the features of every AE report pertaining to Tivozanib. An analysis of disproportionality, a common practice in pharmacovigilance research, was conducted to pinpoint possible links between Tivozanib and all AEs in our study (Lindquist et al., 2000; Guo et al., 2022). The reporting odds ratio (ROR), proportional reporting ratio (PRR), Bayesian confidence propagation neural network (BCPNN), and the multi-item gamma Poisson shrinker (MGPS) represent four key indices, determined through conventional formulas to evaluate possible links between Tivozanib and AEs as showed in Table 1.

**TABLE 1:** Four major algorithms used for signal detection.

Algorithms	Equation	Criteria
ROR	ROR = ad/b/c	lower limit of 95% CI > 1, N≥3
	95%CI = e^ln(ROR)±1.96(1/a+1/b+1/c+1/d)^∧^0.5^	
PRR	PRR = a(c + d)/c/(a+b)	PRR≥2, χ^2^≥4, N≥3
	χ2 = [(ad-bc)^∧^2](a+b + c + d)/[(a+b) (c + d) (a+c) (b + d)]	
BCPNN	IC = log_2_a(a+b + c + d) (a+c) (a+b)	IC025 > 0
	95%CI = E(IC) ± 2V(IC)^∧^0.5	
MGPS	EBGM = a(a+b + c + d)/(a+c)/(a+b)	EBGM05 > 2
	95%CI = e^ln(EBGM)±1.96(1/a+1/b+1/c+1/d)^∧^0.5^	

Notes: Equation: a, number of reports containing both the target drug and target adverse drug reaction; b, number of reports containing other adverse drug reaction of the target drug; c, number of reports containing the target adverse drug reaction of other drugs; d, number of reports containing other drugs and other adverse drug reactions.

Abbreviations: 95% CI, 95% confidence interval; N, the number of reports; χ2, chi-squared; IC, information component; IC025, the lower limit of 95% CI, of the IC; E(IC), the IC, expectations; V(IC), the variance of IC; EBGM, empirical Bayesian geometric mean; EBGM05, the lower limit of 95% CI, of EBGM.

The PRR is a simple disproportionality measure used to detect potential signals of adverse drug reactions. It compares the proportion of a specific adverse event reported for a particular drug to the proportion of the same event reported for all other drugs. If the PRR is significantly greater than 1, it suggests a higher reporting rate for the drug-event combination, which might indicate a possible association. Similar to PRR, ROR is a disproportionality measure that uses logistic regression to calculate the odds of reporting an adverse event with a particular drug compared to all other drugs. It accounts for the total number of reports and can adjust for confounding factors. A ROR greater than 1 suggests a higher likelihood of the event being reported with the drug. BCPNN is a more sophisticated algorithm that uses a Bayesian approach to estimate the probability of a causal relationship between a drug and an adverse event. It can handle sparse data and is less likely to generate false signals compared to PRR. The algorithm propagates the strength of evidence through a network of nodes representing drugs and events, updating the probability of a link based on the evidence from all related nodes. MGPS is a shrinkage method used to adjust the effect sizes (like PRR or ROR) to reduce the number of false-positive signals. It applies a gamma distribution to the observed counts and then shrinks the estimates towards a common value (often zero), which helps in prioritizing the most likely signals. Each of these algorithms has its strengths and limitations and is chosen based on the specific goals of the analysis, the nature of the data, and the need to balance sensitivity and specificity in signal detection. Among the four algorithms, a minimum of one should be identified as a positive indicator of drug-related AEs (lower limit of 95% CI > 1, N≥3; PRR≥2, χ2≥4, N≥3; IC025 > 0 or EBGM05 > 2). In our research, the AE signals that met one of the above four algorithm standards were selected and analyzed for research (Shu et al., 2022a; Shu et al., 2022b; Hou et al., 2022).

Additionally, calculations were made for the duration until the onset and the likelihood of severe outcomes from AEs. Reports containing input errors (EVENT_DT before START_DT), inaccurate date records and absent specific data were omitted before data analysis. The onset time is characterized as the time span from START_DT (start date for Tivozanib use) and EVENT_DT (date of AE occurrence). Weibull distribution can ascertain and forecast the fluctuating rise or fall in risk incidence over time, utilizing scale α) and shape β) as key factors to characterize the Weibull distribution’s form (Mazhar et al., 2021; Cui et al., 2023). The primary severe consequences were life-threatening incidents or those leading to hospitalization, disability, or death. Additionally, instances of severe outcomes linked to drug toxicity were tallied, and the ratio was determined by dividing the count of serious outcomes by the aggregate of reported events. Every aspect of data handling and statistical evaluation utilized R software (version 4.3.1) (Li et al., 2021).

## 3 Results

### 3.1 General characteristics

During the period spanning January 2021 to December 2023, a comprehensive total of 5,361,420 AE reports were diligently submitted to the FAERS database. Among these submissions, a noteworthy subset of 1,366 reports specifically pertained to Tivozanib, as delineated in Figure 1. The descriptive attributes of AE reports concerning Tivozanib are meticulously outlined in Table 2. Over the observed timeframe, the incidence of reported AEs exhibited variability, with the year 2022 witnessing the highest frequency (39.46%), closely followed by 2023 (39.02%). According to the significant proportion of cases lacked precise gender, weight, and age data, we could not perform accurate analysis of the basic characteristics of the population using this drug. The detail of these information was showed in Table 2. Predominantly, reports emanated from the United States (98%), with supplementary contributions from Great Britain (0.9%), Argentina (0.2%), Germany (0.1%), Spain (0.1%), France (0.1%), Austria (0.1%), and Poland (0.1%), predominantly submitted by consumers (81.4%) and healthcare professionals (9.2%).

**TABLE 2:** Clinical characteristics of reports with Tivozanib from the FAERS database.

Characteristics		Case number (n)	Case proportion (%)
	Number of events	1,366	
Gender	Male	20	1.5
	Female	6	0.4
	Missing	1,340	98.1
Weight (kg)	<50	1	0.1
	50–100	11	0.8
	>100	4	0.3
	Missing	1,350	98.8
Age(years)	18–64.9	8	0.6
	65–85	11	0.8
	>85	1	0.1
	Missing	1,346	98.5
Reporter’s Type of Occupation	Consumer	1,112	81.4
	Health profession	126	9.2
	Physician	109	8
	Pharmacist	13	1
	Missing	6	0.4
Reported Countries	United States	1,338	98
	Great Britain	12	0.9
	Argentina	3	0.2
	Germany	2	0.1
	Spain	2	0.1
	France	2	0.1
	Austria	1	0.1
	Poland	1	0.1
	Country Not Specified	5	0.4

### 3.2 Signal detection

**FIGURE 2:**
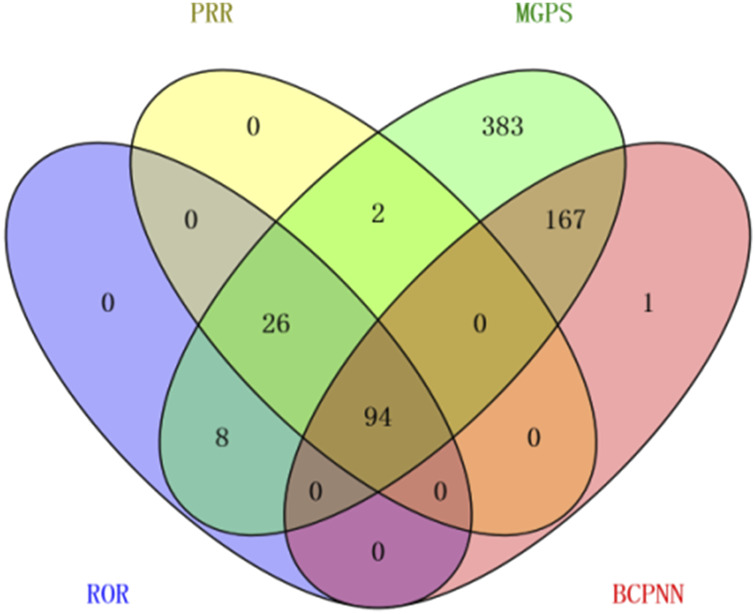
Venn diagram illustrating the overlap of the significant PTs adhering to each aforementioned algorithm.

**FIGURE 3:**
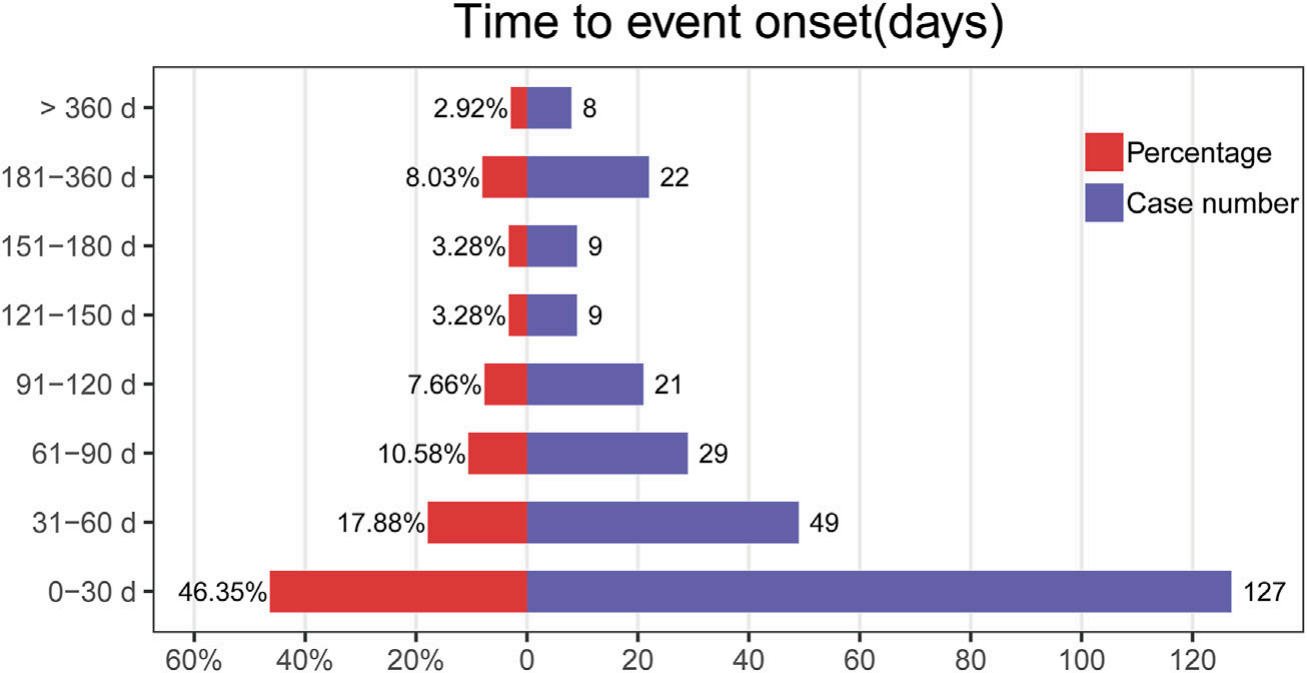
Signal strength of reports of Tivozanib at the preferred terms level in FAERS database.

An exhaustive signal detection analysis unveiled a total of 94 significant PTs of interest conforming to all four algorithms concurrently, with an additional 681 PTs conforming to at least one of the four algorithms (Figure 2, Supplementary Material). Table 3 elaborates on the top 20 PTs, further illustrated in Figure 3. Predominant adverse events, along with their corresponding proportions, encompassed fatigue (7.38%), diarrhea (4.32%), nausea (3.43%), increased blood pressure (3.25%), decreased appetite (3.25%), and dysphonia (2.57%). Notably, these findings align with established instructions and medication warnings. Additionally, unexpected significant AEs, including dyspnea, constipation, extremity pain, stomatitis, and palmar-plantar erythrodysaesthesia syndrome, were discerned.

**TABLE 3:** Signal strength of top 20 AEs of Tivozanib at the preferred terms level in FAERS database.

System organ class (SOC)	Preferred terms	PT (n)	ROR (95% Two-Sided CI)	PRR (95% Two-Sided CI)	χ2	IC(IC250)	EBGM (EBGM05)
Gastrointestinal disorders	Diarrhoea	190	4.3 (3.72–4.98)	4.16 (4.02–4.3)	460.44	2.06 (0.39)	4.16 (3.68)
	Nausea	151	3.2 (2.72–3.76)	3.12 (2.97–3.28)	220.12	1.64 (−0.02)	3.12 (2.72)
	Vomiting	65	2.38 (1.86–3.04)	2.36 (2.12–2.6)	51.23	1.24 (−0.43)	2.36 (1.92)
	Constipation	51	3.42 (2.6–4.51)	3.39 (3.12–3.67)	86.37	1.76 (0.1)	3.39 (2.69)
	Stomatitis	33	7.36 (5.22–10.37)	7.31 (6.97–7.65)	179.59	2.87 (1.2)	7.3 (5.48)
	Abdominal discomfort	32	2.48 (1.75–3.51)	2.47 (2.13–2.82)	28.07	1.3 (−0.36)	2.47 (1.85)
General disorders and administration site conditions	Fatigue	325	5.96 (5.32–6.67)	5.59 (5.49–5.7)	1239.49	2.48 (0.81)	5.58 (5.08)
	Asthenia	79	3.38 (2.71–4.22)	3.34 (3.12–3.56)	129.99	1.74 (0.07)	3.34 (2.77)
	Death	74	1.25 (0.99–1.57)	1.25 (1.02–1.47)	3.67	0.32 (−1.35)	1.25 (1.03)
	Pain	73	1.25 (1–1.58)	1.25 (1.02–1.48)	3.69	0.32 (−1.34)	1.25 (1.03)
Investigations	Blood pressure increased	143	12.59 (10.66–14.88)	12.22 (12.05–12.38)	1471.09	3.61 (1.94)	12.17 (10.59)
	Weight decreased	47	2.37 (1.78–3.16)	2.35 (2.07–2.64)	36.67	1.23 (−0.43)	2.35 (1.85)
Metabolism and nutrition disorders	Decreased appetite	143	9.11 (7.71–10.76)	8.84 (8.68–9)	995.6	3.14 (1.47)	8.82 (7.67)
Musculoskeletal and connective tissue disorders	Pain in extremity	34	1.79 (1.27–2.5)	1.78 (1.44–2.11)	11.66	0.83 (−0.84)	1.78 (1.34)
Nervous system disorders	Headache	47	1.19 (0.89–1.58)	1.19 (0.9–1.47)	1.38	0.25 (−1.42)	1.19 (0.93)
	Dizziness	40	1.34 (0.98–1.83)	1.34 (1.03–1.64)	3.41	0.42 (−1.25)	1.34 (1.03)
Respiratory, thoracic and mediastinal disorders	Dysphonia	113	29.2 (24.2–35.22)	28.47 (28.29–28.65)	2972.5	4.82 (3.15)	28.24 (24.14)
	Dyspnoea	56	1.57 (1.2–2.04)	1.56 (1.3–1.82)	11.32	0.64 (−1.03)	1.56 (1.25)
Skin and subcutaneous tissue disorders	Palmar-plantar erythrodysaesthesia syndrome	33	19.7 (13.97–27.78)	19.56 (19.22–19.9)	578.01	4.28 (2.61)	19.45 (14.59)
Vascular disorders	Hypertension	55	3.86 (2.96–5.03)	3.82 (3.56–4.09)	114.88	1.93 (0.27)	3.82 (3.06)

Abbreviations: ROR, reporting odds ratio; CI, confidence interval; PRR, proportional reporting ratio; χ2, chi-squared; IC, information component; EBGM, empirical Bayesian geometric mean. The words bold in PT (n) were the top 5 significant PT, which resulted from Tivozanib.

**FIGURE 4:**
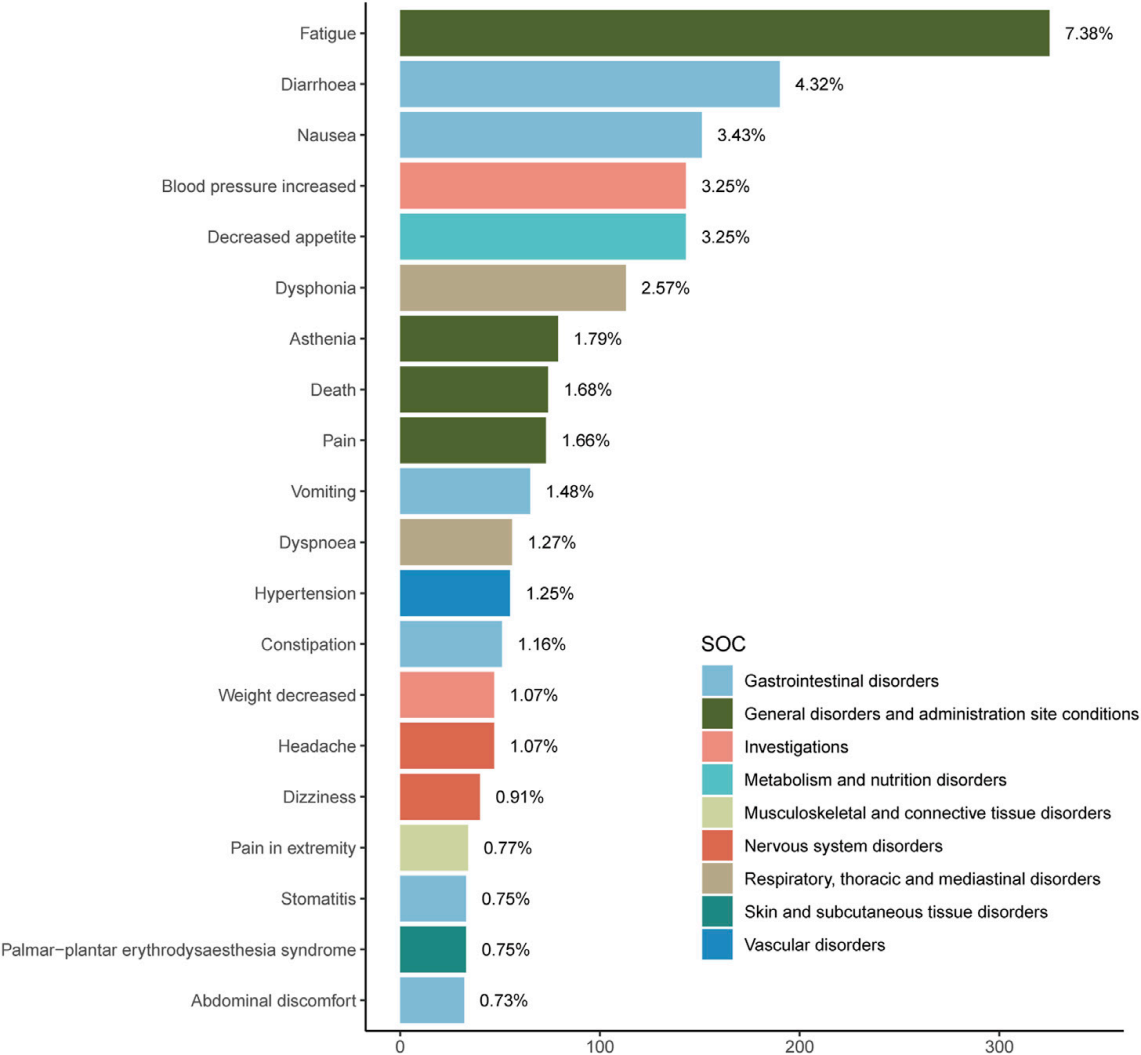
Signal Strength of top20 AEs of Tivozanib at the System Organ Class (SOC) Level in FAERS Database.

Visualization in Figure 4 delineates signal strengths and reporting patterns of Tivozanib at the System Organ Class (SOC) level. Notably, Tivozanib-induced AEs were statistically associated with 26 distinct SOCs, with the top five significant SOCs being “Metabolism and nutrition disorders”, “Gastrointestinal disorders”, “Endocrine disorders”, “Respiratory, thoracic and mediastinal disorders”, and “Surgical and medical procedures".

### 3.3 Time to onset of Tivozanib-Associated Adverse Events

**FIGURE 5:**
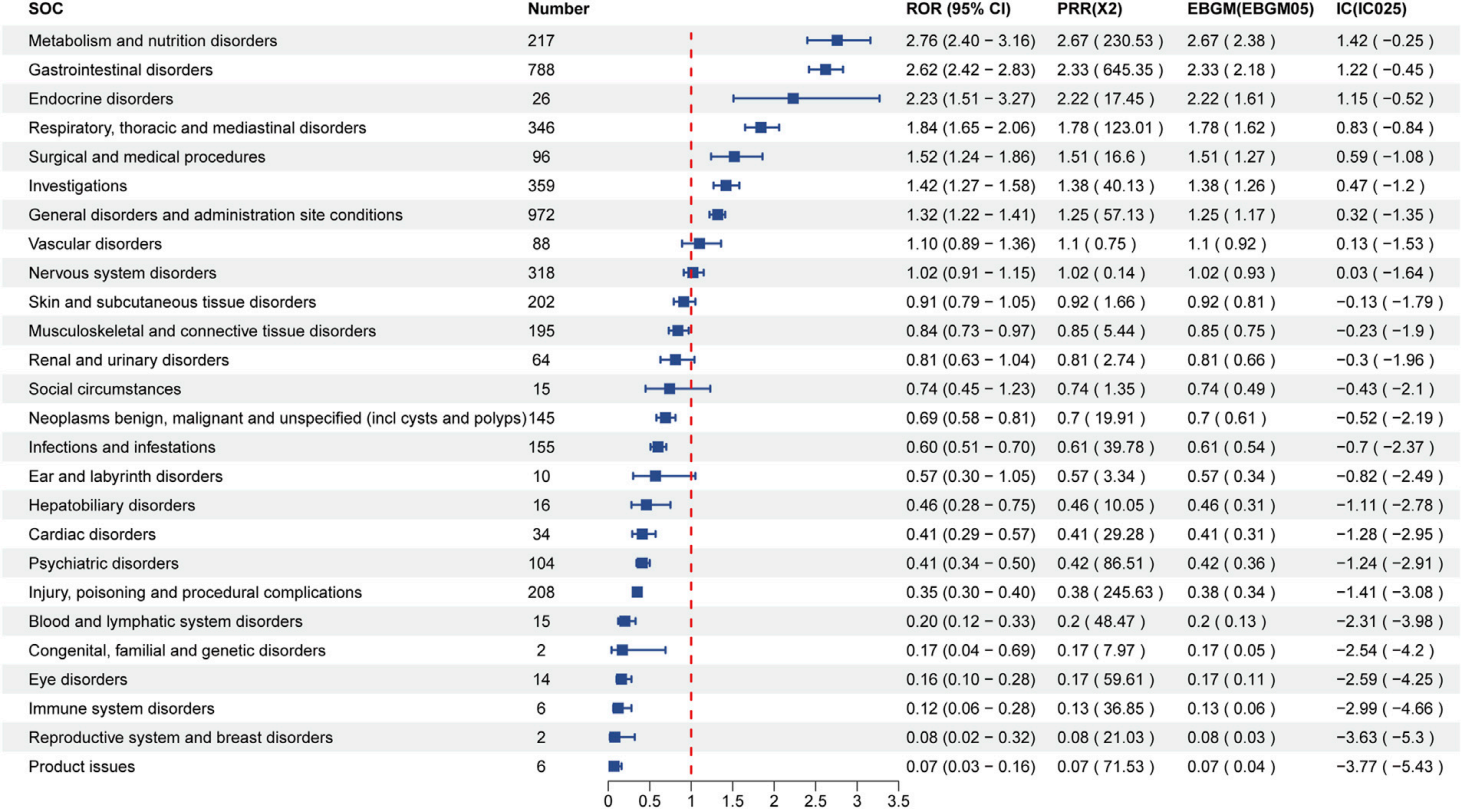
Time to event onset times.

The onset times of Tivozanib-associated AEs were extracted from the database. Excluding inaccurate, missing or unknown onset time reports, a total of 274 Tivozanib-associated AEs reported onset time and the median onset time was 37 days (interquartile range [IQR] 11.75–91 days). As Figure 5 illustrated, results indicated that most of the AE cases occurred within the first (n = 127, 46.35%) and second month (n = 49, 17.88%) after Tivozanib initiation.

Evaluation of the Weibull Shape Parameter analysis (Table 4) revealed a calculated shape parameter β) of 0.45, with a 95% confidence interval (CI) ranging from 0.42 to 0.48. Notably, the obtained value of β indicated a decreasing incidence trend of AEs over time, suggestive of an early failure type.

**TABLE 4:** Time-to-onset analysis for tivozanib-related signals using the Weibull distribution test.

Drug	Parameter	Mean	95% CI	Type
Tivozanib	Median	37	11.75–91	Early failure
	Scale parameter: α	68.92	49.59–88.24	
	Shape parameter: β	0.45	0.42–0.48	

## 4 Discussion

To the best of our knowledge, this is the first most comprehensive and systematic pharmacovigilance study on Tivozanib-associated AEs by post-marketing based on the FAERS database. Our investigation offers a nuanced and meticulous characterization of Tivozanib-related AEs, providing a more precise understanding of their nature and prevalence.

As a potent VEGF tyrosine kinase inhibitor, Tivozanib exerts its action primarily through inhibition of VEGF receptors 1 and 2 (Cowey, 2013; Jacob et al., 2020). Given the pivotal role of VEGF in both physiological and pathological angiogenic processes, the use of anti-VEGF agents in oncology is accompanied by a spectrum of side effects stemming from the disruption of normal VEGF-mediated vascular integrity (Melincovici et al., 2018; Porta and Striglia, 2020; Alanazi et al., 2023). Our analysis underscores the clinical relevance of this mechanism, particularly evident in the frequent occurrence of elevated blood pressure among the study cohort, with approximately 143 cases documenting this adverse event. Furthermore, our disproportionality analysis conducted at the SOC level highlights the presence of vascular and cardiac disorders among the list of significant SOCs, findings that align with safety data documented in product labeling and clinical trials.

In our analysis of PT levels within the results, gastrointestinal disorders, encompassing symptoms such as diarrhea, nausea, vomiting, constipation, stomatitis, abdominal discomfort, prominently featured as the most prevalent AEs. This pattern of gastrointestinal AEs is in line with observations commonly associated with chemotherapy drugs. Additionally, a spectrum of other disorders emerged, including general disorders and administration site conditions (fatigue, asthenia, pain, and death), investigations (increased blood pressure, decreased weight), metabolism and nutrition disorders (decreased appetite), musculoskeletal and connective tissue disorders (extremity pain), nervous system disorders (headache, dizziness), respiratory, thoracic, and mediastinal disorders (dysphonia, dyspnea), and skin and subcutaneous tissue disorders (palmar-plantar erythrodysaesthesia syndrome), albeit in partial instances. These findings underscore the potential risks associated with Tivozanib administration and emphasize the necessity of cautious patient management strategies. Inevitably, we cannot distinguish whether these adverse reactions are caused by combination therapy, underlying health conditions, or treatment strategies. Therefore, more experiments need to be conducted to further validate the existing conclusions.

By analyzing the time to onset, researchers and healthcare professionals can better understand the safety profile of a drug, which is crucial for patient education and informed consent. Besides, analyzing the time to onset could evaluate risk of causing AEs within specific timeframes. Some side effects might be more likely to occur early in the treatment, while others might emerge after prolonged use. Time to onset data also can inform post-marketing surveillance efforts, helping to detect previously unrecognized risks or to confirm the known safety profile of a drug. In the drug development phase, understanding the time to onset can inform the design of clinical trials and the interpretation of results, particularly in the context of dose-finding and safety studies. It aids in communicating with patients about what to expect in terms of side effects and when they are likely to occur, which can improve patient compliance and satisfaction. Clinicians may use this information to make decisions about continuing, modifying, or discontinuing a treatment regimen based on the risk-benefit analysis considering the time to onset of AEs. Our study revealed that the median onset time for Tivozanib-associated AEs was determined to be 37 days, with a notable concentration of cases occurring within the initial 1–2 months following Tivozanib initiation. According to the median onset time for Tivozanib-associated AEs, more attention should be taken on the patients after 1 month. And the medication method and dosage need to be adjusted in a timely manner to avoid adverse reactions. Consequently, prolonged monitoring periods are warranted to comprehensively assess and observe ADRs associated with Tivozanib in forthcoming clinical investigations.

In the analysis of adverse drug reactions, “time to onset” refers to the period that elapses between the initiation of drug treatment and the appearance of an adverse event or side effect. Statistical power in the analysis of Time to Onset of drug-Associated Adverse Events refers to the probability that a study will detect an effect when there is a true effect present. It is a critical measure of the sensitivity of a statistical test to detect a difference or an effect if it exists. In the context of clinical trials and the analysis of AEs associated with a drug, high statistical power is desirable as it minimizes the chance of a Type II error, which is failing to detect a true effect. In practice, statistical power is often calculated *a priori*, before data collection begins, to determine the required sample size to detect an effect of a certain size with a given level of confidence. Post hoc power calculations can also be performed after data collection to assess the power of the study based on the observed effect size. It is important to note that while high statistical power is beneficial, it must be balanced against other considerations such as the cost and practicality of increasing sample size, the potential for subject burden, and ethical considerations. Additionally, a focus on statistical power should not overshadow the importance of clinical significance and the interpretation of study results in the context of existing evidence.

Based on the adverse events related to Tivozanib and the timing of their onset obtained in this study, we propose the following specific clinical recommendations. Firstly, optimizing clinical monitoring strategies is necessary, with a focus on early detection and management of AEs. Physicians should consider more frequent monitoring of vital signs and blood parameters, especially within the initial month following the initiation of Tivozanib therapy, during which the majority of AEs are observed. Secondly, proactive measures for AE prevention and management are crucial. Adjusting dosage regimens, considering combination therapies, or implementing symptomatic treatment strategies can alleviate the impact of Tivozanib-related AEs. Furthermore, patient education plays a vital role in improving treatment adherence and early AE recognition. Providing comprehensive information about potential adverse events and encouraging open communication between patients and healthcare providers is essential. Moreover, individualized treatment strategies based on patient-specific factors and the severity of AEs are paramount. Customizing treatment plans to minimize the impact of adverse reactions on patients’ quality of life requires a personalized approach, balancing therapeutic efficacy and safety considerations. In conclusion, our study not only contributes to the understanding of Tivozanib-related AEs but also emphasizes the importance of translating these findings into actionable clinical recommendations. By optimizing monitoring strategies, implementing preventive measures, and promoting patient-centered care, we can enhance the safety and effectiveness of Tivozanib therapy in clinical practice.

In our investigation, we conducted data retrieval and subsequent statistical analysis from the FAERS utilizing a set of predetermined keywords, including “Tivozanib”, “AV951”, “AV951 CPD”, “Tivozanib capsule”, “AV-951”, “Fotivda”, “Tivozanib hydrochloride”, “KRN951”, “KRN 951”, and “KRN-951”. This meticulous approach aimed to mitigate potential analysis inaccuracies arising from incomplete dataset (Haberkorn and Eskens, 2013; Passi et al., 2023). Nonetheless, notable inconsistencies or omissions persisted in essential patient demographics such as age, weight, and gender. Such data incompleteness introduces the possibility of analysis bias, impeding the precise identification of the most appropriate and responsive target audience for assessing drug efficacy. Notably, a predominant representation of cases was from the United States (98%) while only few reports from other countries were obtained in our study, which will complicate the generalization of our conclusions to broader populations.

In the context of FAERS data analysis, researchers often use disproportionality measures such as ROR, PRR and BCPNN, which can help detect signals even with lower numbers of reports. However, it is important to acknowledge the limitations of FAERS when interpreting the results in terms of statistical power. When discussing the statistical power, it is also beneficial to reference the specific criteria used to determine a signal as significant. For instance, a positive signal might be defined by a combination of a minimum number of reports, a certain threshold for the ROR, and a *p*-value from a χ2 test. These criteria are designed to balance the need for a sufficiently powered study against the reality of data sparsity in FAERS.

Despite the advantages conferred by expansive real-world large-sample research and the data mining techniques employed herein, certain limitations persist, warranting careful consideration (Nguyen et al., 2012; Moja et al., 2015). Primarily, the spontaneous reporting mechanism inherent to FAERS may lead to the accumulation of partial and inaccurate data from disparate sources globally, potentially compromising data quality and introducing analytical biases. For instance, the most reports in our study were from the United States so that the results of our analysis might occur differently in other regions. These issues suggest that a more comprehensive database should be established in the future to obtain more complete and detailed clinical medication data. Moreover, unaccounted variables such as drug interactions, concurrent medical conditions, and the concomitant use of multiple medications, which could impact adverse event profiles, were not systematically incorporated into our analysis. Furthermore, while FAERS offers an extensive repository of case reports, it inherently lacks comprehensive details on patients who have utilized the drug without experiencing adverse events, thereby hindering the establishment of a definitive causal relationship. Notably, the disproportionality analysis conducted herein, while indicative of signal strength, did not ascertain risk or establish causality but rather inferred statistical significance of observed signals. Hence, there remains a critical imperative for prospective clinical investigations aimed at elucidating causal links. Despite these constraints, our findings serve as a vital resource for medical practitioners to vigilantly monitor patients and ascertain the associated adverse effects of Tivozanib (Shu et al., 2022a; Shu et al., 2022b).

## 5 Conclusion

In summary, our pharmacovigilance analysis of the FAERS database has scientifically and systematically quantified the potential risks associated with Tivozanib treatment, including the time to onset of AEs and the spectrum of safety signals. Notably, our study has identified unexpected and novel significant AEs, such as dyspnea, constipation, pain in extremity, stomatitis, and palmar-plantar erythrodysaesthesia syndrome, which manifest with notable frequency and warrant close patient monitoring. However, the limitations in data completeness and potential biases inevitably affects the reliability and applicability of our findings. The acquisition of complete information will enhance the possibility of excluding drug interactions and atypical adverse reactions caused by personal characteristics of patients and personalized treatment. The insights gleaned from our research offer valuable evidence to guide further investigations and inform clinical practice regarding the use of Tivozanib.

## Data Availability

The original contributions presented in the study are included in the article/Supplementary Material, further inquiries can be directed to the corresponding author.
